# Neonatal Total Parenteral Nutrition and Long-Term Metabolic Outcomes: An 18-Year Nationwide Cohort Study of 2.3 Million Infants

**DOI:** 10.3390/nu18152406

**Published:** 2026-07-23

**Authors:** Tak Kyu Oh, In-Ae Song

**Affiliations:** 1 Department of Anesthesiology and Pain Medicine, Seoul National University Bundang Hospital, Seongnam 13620, Republic of Korea; 66034@snubh.org; 2 Department of Anesthesiology and Pain Medicine, Seoul National University College of Medicine, Seoul 03080, Republic of Korea

**Keywords:** total parenteral nutrition, neonatal nutrition, type 2 diabetes mellitus, metabolic programming

## Abstract

**Background/Objectives**: Total parenteral nutrition (TPN) is essential for neonates unable to receive adequate enteral feeding, but its long-term metabolic effects remain uncertain. This study evaluated associations between neonatal TPN exposure, exposure duration, and later endocrinologic or metabolic disorders. **Methods**: This nationwide cohort included 2,424,716 infants born in South Korea between 2005 and 2010. TPN exposure was identified during the first 28 days of life. The primary outcome was the first occurrence of type 2 diabetes mellitus (T2DM), essential hypertension, dyslipidemia, or obesity during follow-up of up to 18 years. The primary analysis used inverse probability of treatment-weighted (IPTW) Cox models, whereas duration-based associations were evaluated using secondary exploratory multivariable Cox regression. **Results**: The final cohort included 2,303,567 infants, of whom 7656 received TPN. In the primary IPTW analysis, any neonatal TPN exposure was not associated with either the primary composite outcome (HR 1.01, 95% CI 0.94–1.09; *p* = 0.801) or T2DM (HR 1.12, 95% CI 0.93–1.35; *p* = 0.215). In secondary exploratory multivariable analyses, TPN exposure for ≥3 days was associated with T2DM (adjusted HR 2.14, 95% CI 1.34–3.41; *p* = 0.001), whereas exposure for 1–2 days was not. **Conclusions**: The primary IPTW analysis showed no significant association between any neonatal TPN exposure and the primary composite outcome or T2DM. The association observed for ≥3 days in secondary exploratory analyses should be regarded as hypothesis-generating.

## 1. Introduction

Adequate nutritional support during the neonatal period, particularly for preterm or critically ill infants, is a fundamental prerequisite for survival and optimal neurodevelopmental outcomes [1]. For infants unable to tolerate enteral feeding, total parenteral nutrition (TPN) has become an indispensable, life-saving intervention in clinical practice [2]. TPN provides not only calories but also key macro- and micronutrients, including glucose, amino acids, lipid emulsions, electrolytes, vitamins, minerals, and trace elements, during a highly sensitive developmental period [1,2].

Emerging evidence from the Developmental Origins of Health and Disease framework suggests that early-life nutritional exposures can shape later metabolic health [3,4,5]. Direct intravenous nutrient delivery bypasses the gastrointestinal tract during a period of rapid endocrine and metabolic maturation. This non-physiological route may alter enteral nutrient sensing, incretin signaling, insulin secretion, protein metabolism, hepatic fatty acid handling, and oxidative stress responses [6,7,8,9].

The physiological concern regarding TPN stems from its circumvention of the natural enteral route, which may attenuate gut-mediated incretin signaling involved in insulin secretion [8]. Intravenous glucose and lipid infusions may induce abnormal insulin responses and increase metabolic stress on developing pancreatic beta-cells [7,9]. During early life, these effects may influence metabolic programming through persistent changes in beta-cell function, insulin sensitivity, adiposity, hepatic lipid metabolism, and epigenetic regulation [3,4,5,7,9,10].

However, human evidence remains limited. Previous studies have often been constrained by small sample sizes, short follow-up periods, or a focus on short-term growth and neurodevelopmental outcomes rather than late-onset metabolic disease [11,12]. Despite the theoretical importance of critical developmental windows, it remains unclear whether neonatal TPN exposure has a duration-dependent association with long-term endocrinologic or metabolic disorders.

This nationwide population-based cohort study aimed to evaluate the association between neonatal TPN exposure and long-term endocrinologic or metabolic outcomes over an 18-year follow-up period. The study specifically examined whether this association differed according to TPN duration and whether a clinically meaningful exposure threshold could be identified. By using a national birth cohort of more than 2.3 million infants, this study provides one of the largest and longest human evaluations of neonatal TPN exposure and subsequent metabolic health.

## 2. Materials and Methods

### 2.1. Study Design and Ethical Statement

This population-based, retrospective cohort study was conducted using national administrative healthcare data. The study protocol was developed in strict accordance with the Strengthening the Reporting of Observational Studies in Epidemiology (STROBE) statement to ensure methodological rigor [13]. The Institutional Review Board of Seoul National University Bundang Hospital (No. X-2411-939-903; approval date: 13 November 2024) and the National Health Insurance Service (NHIS) (No. NHIS-2025-02-1-010) provided ethical approval for this research. In compliance with the Declaration of Helsinki and national regulations, the requirement for informed consent was waived, as the analysis utilized fully de-identified and anonymized data routinely collected for clinical and billing purposes.

### 2.2. Data Source

Individual-level data were retrieved from the NHIS database, a comprehensive repository encompassing the entire South Korean population under a compulsory, universal single-payer system. This longitudinal dataset provides longitudinal records of sociodemographic factors, medical procedures, pharmaceutical prescriptions, and mortality. Diagnostic classifications were defined according to the International Classification of Diseases, 10th Revision (ICD-10). While the majority of healthcare services in South Korea are delivered by private providers, the data maintain high integrity and consistency due to the government’s centralized monitoring of all medical billing and reporting practices [14].

### 2.3. Study Population

A nationwide birth cohort was established comprising all neonates born in South Korea between 1 January 2005, and 31 December 2010. To investigate the long-term metabolic consequences of early nutritional interventions, infants were categorized into two groups based on their exposure to TPN during the neonatal period (the first 28 days of life). The TPN group included neonates who received at least one prescription of parenteral nutrition, while the control group consisted of those with no record of TPN administration.

Neonatal TPN exposure was identified using prescription and procedure claims for parenteral nutrition recorded during the first 28 days of life. TPN duration was calculated as the total number of days with recorded TPN administration during this neonatal period. Infants without any TPN record were assigned a TPN duration of 0 days. Because the NHIS database is an administrative claims database, detailed information on total energy intake, individual nutrient contributions, glucose infusion rate, amino acid dose, lipid emulsion type or dose, micronutrient composition, and specific TPN formula was not available. Therefore, the present study evaluated TPN exposure and duration rather than energy intake, nutrient dose, or formula-specific effects.

Several exclusion criteria were applied to reduce potential confounding from pre-existing conditions. Neonates diagnosed with chromosomal abnormalities or inborn errors of metabolism (ICD-10 codes E70–E88 and Q90–Q99) were excluded. Additionally, to focus on long-term outcomes and mitigate immortal time bias, infants who died within the first year of life were removed from the analysis.

### 2.4. Study Endpoint

The primary endpoint of this study was the first occurrence of any endocrinologic or metabolic disorder during the follow-up period. This composite outcome was defined by the presence of at least one of the following diagnostic codes according to the ICD-10: T2DM (E11), essential hypertension (I10), disorders of lipoprotein metabolism and other lipidemias (E78), or obesity (E66). Metabolic syndrome was operationally defined as the occurrence of two or more distinct metabolic components among T2DM, essential hypertension, dyslipidemia, and obesity in a single participant during longitudinal follow-up. This definition was employed to capture the clustering of metabolic risk factors, reflecting a more severe state of metabolic derangement than a single-organ disorder.

Secondary endpoints included the individual evaluation of each component of the composite outcome. Among these, T2DM (E11) was designated as a key secondary endpoint of particular clinical interest, given its established link to early-life nutritional programming.

For each individual endpoint, the date of diagnosis was defined as the first recorded claim with the relevant ICD-10 code in either inpatient or outpatient settings. For the composite outcome, the event date was defined as the earliest diagnosis date of any component disorder. For metabolic syndrome, the event date was defined as the date on which a second distinct metabolic component was first documented. To ensure the robustness of the results and exclude transient neonatal physiological variations, only diagnoses documented after the first year of life were included. Participants were censored at the time of their initial diagnosis, death, or the study’s conclusion on 31 December 2023, whichever occurred first.

### 2.5. Covariates

To mitigate potential confounding and enhance the robustness of our findings, a comprehensive set of maternal and neonatal characteristics was incorporated into the analytical models. These included maternal socioeconomic profiles, pre-existing neuropsychiatric history, and obstetric complications, alongside neonatal clinical status and relevant medical or surgical interventions. Neonatal covariates were ascertained from diagnostic and procedure claims recorded during the first 28 days of life. Because TPN exposure was assessed during the same interval, these covariates were not necessarily restricted to events occurring before TPN initiation and were therefore interpreted as neonatal clinical severity markers rather than strictly pre-exposure baseline confounders. Comprehensive descriptions of these covariates and their corresponding ICD-10 codes are documented in Supplemental File S1.

### 2.6. Statistical Analysis

Our statistical approach comprised two complementary phases designed to rigorously validate the stability and reproducibility of the observed associations. To address potential confounding by indication—a critical concern in neonatal nutritional studies—the primary analysis utilized inverse probability of treatment weighting (IPTW) [15]. Propensity scores, representing the conditional probability of neonatal TPN exposure, were derived from a multivariable logistic regression model incorporating the maternal characteristics and neonatal clinical severity markers described above. We applied weights based on the average treatment effect on the treated (ATT) to focus the inference specifically on the TPN-exposed population. Covariate balance before and after weighting was assessed using absolute standardized mean differences (SMDs). Consistent with the ATT estimand, SMDs were standardized using the unweighted standard deviation of the TPN-exposed group as the denominator. For binary covariates, standardized differences rather than raw differences in proportions were reported. An absolute SMD of <0.10 was considered to indicate adequate balance, whereas an SMD of ≥0.10 indicated residual imbalance. The effective sample size after weighting was also calculated. Weighted Cox proportional hazards regression models were used to estimate hazard ratios (HRs) and 95% confidence intervals (CIs) for the primary and secondary outcomes.

In a sensitivity analysis, multivariable Cox proportional hazards regression was performed on the entire unweighted cohort to verify the consistency of the IPTW findings across different modeling frameworks. Because the primary IPTW estimand compared any TPN exposure with no exposure, TPN duration categories were evaluated as secondary exploratory exposures using covariate-adjusted Cox regression in the full cohort rather than separate duration-specific weighted models. To ensure the stability of these models, multicollinearity was evaluated using variance inflation factors (VIF), with a value < 5 signifying the absence of significant collinearity. Model parsimony and fit were examined via likelihood ratio tests and Wald statistics. The proportional hazards assumption was empirically validated through the inspection of Schoenfeld residuals.

As secondary exploratory analyses, TPN duration was categorized into 1–2 days and ≥3 days of exposure. Because detailed energy and nutrient composition data were unavailable, dose–response analyses were based on the duration of recorded TPN exposure rather than cumulative caloric or nutrient dose. Subgroup analyses were further conducted to identify potential effect modification by clinical severity markers, including mechanical ventilation support, neonatal intensive care unit (NICU) admission, and low birth weight.

As an exploratory graphical analysis, a Spearman correlogram was constructed using the unweighted raw cohort to visualize pairwise correlations between neonatal TPN duration and long-term endocrinologic or metabolic outcomes, including T2DM, essential hypertension, dyslipidemia, obesity, metabolic syndrome, and the composite outcome. This analysis was interpreted descriptively to illustrate the crude correlation structure among TPN duration and metabolic outcomes.

All analyses were conducted using R software (version 4.3.1; R Foundation for Statistical Computing, Vienna, Austria). Propensity score weighting was implemented using the ‘WeightIt’ package, while weighted and multivariable survival models were constructed via the ‘survey’, ‘survival’, and ‘car’ packages. The correlogram was generated using the ‘corrplot’ package. All tests were two-sided, and a *p*-value < 0.05 was considered statistically significant.

## 3. Results

### 3.1. Study Population and Baseline Characteristics

**Figure 1:**
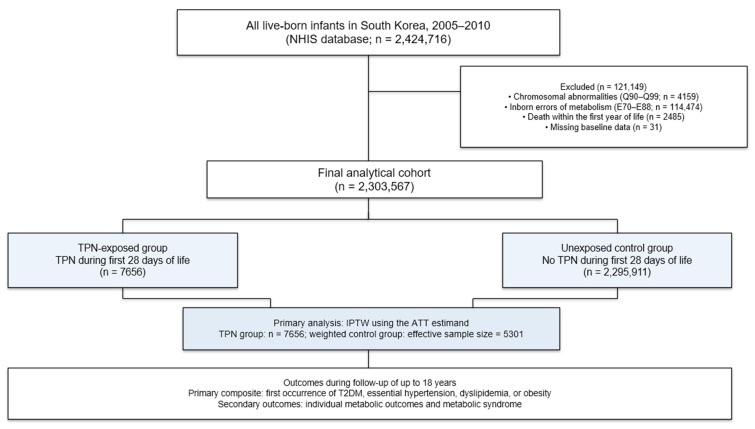
Study flow diagram and analytical framework. TPN: Total Parenteral Nutrition, IPTW: Inverse Probability of Treatment Weighting.

The detailed participant selection process, including specific exclusion criteria and the final distribution of the study groups, is illustrated in Figure 1. From an initial screening of 2,424,716 neonates born between 2005 and 2010, a final cohort of 2,303,567 infants met the eligibility criteria and were included in the longitudinal analysis. Of these, 7656 (0.3%) infants were exposed to TPN during the neonatal period, while 2,295,911 (99.7%) served as the control group.

Baseline characteristics of the study population—encompassing maternal sociodemographics, neuropsychiatric history, obstetric complications, and neonatal clinical factors—are compared between the groups in Table 1. Prior to adjustment, the TPN group exhibited significantly higher clinical severity and neonatal morbidity than the control group. Specifically, infants in the TPN group were substantially more likely to be born preterm or with low birth weight (76.8% vs. 2.4%; SMD 1.763), require mechanical ventilation (66.2% vs. 1.0%; SMD 1.380), and have a higher prevalence of bacterial sepsis (47.2% vs. 2.5%; SMD 0.895). Differences were also observed in several maternal and obstetric characteristics.

Following the application of IPTW to estimate the ATT, the effective sample size of the weighted control group was 5301. Covariate balance was substantially improved after weighting, particularly for maternal sociodemographic characteristics, obstetric factors, and birth year. However, residual imbalance, defined as an absolute SMD of ≥0.10, remained for eight neonatal covariate levels, with post-weighting SMDs ranging from 0.110 to 0.242. The largest residual imbalance was observed for congenital gastrointestinal malformations (SMD, 0.242). Other covariates with residual imbalance included low birth weight or preterm birth, bacterial sepsis, necrotizing enterocolitis, mechanical ventilation, congenital circulatory malformations, and the pediatric complex chronic condition categories of 0 and ≥2. Thus, IPTW markedly reduced, but did not completely eliminate, differences in neonatal illness severity between the groups.

### 3.2. Primary and Secondary Outcomes

During the 18-year follow-up period, neonatal TPN exposure was not significantly associated with the primary composite outcome of endocrinologic and metabolic disorders in the primary IPTW-adjusted analysis (Weighted HR 1.01, 95% CI 0.94–1.09, *p* = 0.801; Table 2). Analysis of individual secondary outcomes similarly showed no significant risk for essential hypertension (Weighted HR 0.95, 95% CI 0.68–1.31, *p* = 0.733), dyslipidemia (Weighted HR 0.98, 95% CI 0.91–1.06, *p* = 0.615), or obesity (Weighted HR 1.02, 95% CI 0.75–1.39, *p* = 0.900). While the risk of T2DM appeared numerically higher in the TPN group, the association did not reach statistical significance in the overall cohort (Weighted HR 1.12, 95% CI 0.93–1.35, *p* = 0.215).

### 3.3. Secondary Exploratory Analyses According to TPN Duration

In secondary exploratory multivariable analyses of the full unweighted cohort, TPN exposure for ≥3 days was associated with an increased risk of T2DM compared with no TPN exposure (adjusted HR 2.14, 95% CI 1.34–3.41; *p* = 0.001; Table 3), whereas exposure for 1–2 days was not associated with T2DM. The association between TPN exposure for ≥3 days and the primary composite outcome did not reach conventional statistical significance (adjusted HR 1.29, 95% CI 1.00–1.67; *p* = 0.055). These duration-based findings should be considered hypothesis-generating and do not establish a causal dose–response relationship or a biological threshold at 3 days. All HRs and 95% CIs from the multivariable Cox regression models are presented in Supplementary File S2. The exploratory Spearman correlogram of TPN duration and metabolic outcomes is presented in Figure S1.

### 3.4. Subgroup and Sensitivity Analyses

In exploratory subgroup analyses (Table 4), TPN exposure for ≥3 days was associated with the primary composite outcome among infants requiring mechanical ventilation (adjusted HR 1.46, 95% CI 1.11–1.92; *p* = 0.006), infants with low birth weight or preterm birth (adjusted HR 1.59, 95% CI 1.19–2.12; *p* = 0.002), and infants admitted to the NICU (adjusted HR 1.60, 95% CI 1.21–2.12; *p* = 0.001). Because these analyses were secondary and involved clinically selected subgroups, the findings should be interpreted as exploratory and may reflect residual differences in neonatal illness severity. In sensitivity analyses of the full unweighted cohort, multivariable Cox regression yielded findings broadly consistent with the primary IPTW analysis for any TPN exposure, with no significant association with either the primary composite outcome or T2DM.

## 4. Discussion

In this large-scale, population-based cohort study of more than 2.3 million infants with an 18-year follow-up, our findings provide a generally reassuring message regarding the long-term metabolic safety of neonatal TPN. After rigorous adjustment for maternal and neonatal covariates using IPTW, neonatal TPN exposure was not associated with a significantly increased risk of the primary composite outcome, which included T2DM, essential hypertension, dyslipidemia, and obesity (HR 1.01, 95% CI 0.94–1.09, *p* = 0.801). Moreover, TPN exposure for 1–2 days was not associated with any of the evaluated metabolic outcomes. Together, these findings suggest that brief, clinically indicated TPN exposure is unlikely to substantially increase long-term metabolic risk for most infants and support its continued use as an essential bridge to enteral nutrition when enteral feeding is not feasible [2,6].

However, an important finding was the duration-dependent association between neonatal TPN and later metabolic risk. TPN exposure for 3 days or longer was associated with a more than two-fold increase in the risk of T2DM (HR 2.14, 95% CI 1.34–3.41, *p* = 0.001). This association was more pronounced among clinically vulnerable infants, including those requiring mechanical ventilation, those born with low birth weight, and those admitted to the NICU. Nevertheless, the ≥3-day exposure category should not be interpreted as a definitive biological cutoff or as evidence that TPN itself caused subsequent T2DM. Rather, prolonged TPN duration may reflect the combined effects of cumulative parenteral nutrient exposure, greater neonatal illness severity, feeding intolerance, and delayed transition to enteral nutrition. The identified duration category should therefore be regarded as a clinically relevant risk marker that warrants further investigation.

Several biological mechanisms may explain the observed association between prolonged neonatal TPN exposure and later T2DM. A central hypothesis involves the bypass of the entero-insular axis. Under physiological enteral feeding, nutrient exposure stimulates the release of incretin hormones, including glucagon-like peptide-1, which augment glucose-dependent insulin secretion [8]. Because TPN bypasses gastrointestinal nutrient sensing, it may attenuate incretin-mediated signaling and expose immature pancreatic beta-cells to a non-physiological pattern of intravenous glucose delivery. Repeated glycemic excursions and increased insulin demand during a critical period of pancreatic development may influence beta-cell maturation, functional beta-cell mass, and subsequent insulin sensitivity [8,16]. Experimental evidence also supports the possibility that neonatal parenteral nutrition can induce persistent alterations in insulin signaling and glucose metabolism [7,9].

Parenteral nutrient delivery may also affect long-term metabolic health through changes in protein metabolism, growth, and hepatic lipid handling. Amino acid provision is essential for neonatal protein accretion, lean tissue development, and catch-up growth; however, the metabolic effects of amino acids depend on the balance between nutrient supply, growth requirements, and the infant’s capacity for utilization [2,6]. Non-physiological or excessive substrate delivery may alter insulin and insulin-like growth factor signaling, potentially affecting later body composition and insulin sensitivity. Similarly, intravenous lipid emulsions are delivered directly to the systemic circulation and liver, where they may influence hepatic fatty acid uptake, oxidation, triglyceride synthesis, and lipoprotein metabolism during a period of hepatic immaturity [7,9]. These mechanisms may be relevant to the observed associations with T2DM and metabolic syndrome, even in the absence of a clear association with obesity alone.

Furthermore, the duration-dependent association may involve cumulative oxidative and inflammatory stress. TPN formulations, particularly lipid-containing solutions, may generate reactive oxygen species and promote inflammatory signaling [7]. Neonates have relatively immature antioxidant defenses, which may increase their susceptibility to oxidant exposure. Persistent oxidative stress may alter hepatic and pancreatic gene regulation through epigenetic mechanisms and thereby influence glucose and lipid metabolism later in life [3,5,7,10]. However, because information on the actual energy and nutrient composition of TPN was unavailable in this study, the relative contributions of glucose, amino acids, lipid emulsions, and micronutrients could not be determined. These biological explanations should therefore be considered plausible hypotheses rather than mechanisms directly demonstrated by the present data.

Our findings build upon and extend previous clinical investigations, most notably the PEPaNIC randomized controlled trial [11]. The 4-year follow-up of PEPaNIC suggested that withholding early parenteral nutrition in critically ill children did not adversely affect neurodevelopment and may provide some clinical benefits. The present study extends this evidence by evaluating metabolic outcomes through adolescence and early adulthood in a nationwide neonatal cohort. The association between TPN exposure for 3 days or longer and later T2DM may not have been detectable during the shorter follow-up periods of previous studies. This finding supports the possibility that some consequences of early nutritional exposure remain clinically silent for many years before manifesting as overt metabolic disease. Nevertheless, differences in population, clinical setting, exposure definition, and study design preclude direct comparison between the two studies.

From a clinical perspective, these findings do not argue against neonatal TPN or support its arbitrary discontinuation at 3 days. TPN remains essential when adequate enteral nutrition cannot be achieved. Instead, the findings support careful daily reassessment of the ongoing indication for TPN, advancement of enteral feeding when clinically feasible, and individualized prescription of energy and nutrients according to growth requirements and metabolic tolerance. Avoidance of cumulative overfeeding and monitoring of glucose, triglycerides, and hepatic function may be particularly relevant in infants requiring prolonged TPN. Infants with prolonged exposure and markers of neonatal clinical severity may also warrant closer assessment of growth and cardiometabolic health during long-term follow-up.

### Strengths, Limitations, and Future Directions

This study has several strengths. First, the nationwide cohort included more than 2.3 million infants and provided follow-up for up to 18 years, allowing evaluation of metabolic disorders that may not become apparent during early childhood. Second, the large sample size enabled secondary exploratory analyses according to TPN duration and analyses of clinically vulnerable subgroups. Third, IPTW targeting the average treatment effect on the treated was used to improve comparability between exposed and unexposed infants across a broad range of measured maternal and neonatal characteristics, and covariate balance was explicitly assessed using standardized mean differences.

Several limitations should be acknowledged. First, detailed information on energy intake and individual nutrient contributions was unavailable in the NHIS claims database. Consequently, total caloric intake, glucose infusion rate, amino acid dose, lipid emulsion type and dose, micronutrient composition, and institutional or temporal differences in TPN formulas could not be evaluated. The findings therefore reflect associations with recorded TPN duration rather than the effects of specific energy or nutrient components.

Second, residual confounding by indication remains possible despite IPTW. Infants receiving prolonged TPN are likely to have had greater illness severity, gastrointestinal dysfunction, feeding intolerance, or delayed enteral feeding, and these factors may themselves influence later metabolic health. Although IPTW substantially improved covariate balance, residual imbalance remained for several neonatal illness-severity markers, with post-weighting absolute SMDs ranging from 0.110 to 0.242. Therefore, the weighted estimates may still be affected by residual confounding related to neonatal illness severity.

In addition, several neonatal covariates, including sepsis, mechanical ventilation, necrotizing enterocolitis, and NICU-related clinical factors, were identified during the neonatal period and were not necessarily restricted to events occurring before the initiation of TPN. Consequently, the temporal ordering between TPN initiation and some neonatal covariates could not always be established. Some of these variables may therefore represent consequences or intermediates of the underlying illness or treatment rather than true baseline confounders, raising the possibility of overadjustment or collider bias.

Third, outcomes were identified from ICD-10 claims rather than laboratory measurements or standardized clinical assessments. Data on glucose, insulin, lipid profiles, blood pressure measurements, anthropometric trajectories, and biochemical criteria for metabolic syndrome were unavailable. Fourth, post-discharge nutrition, physical activity, socioeconomic changes, and familial metabolic risk could not be fully measured. In addition, the exclusion of infants who died during the first year limits interpretation of the findings to neonatal survivors. Finally, the study was conducted in a South Korean population, and external validation is required before the findings can be generalized to other populations and healthcare systems.

The duration-based findings should also be interpreted cautiously because they were obtained from secondary exploratory multivariable analyses rather than from the primary IPTW analysis. The primary IPTW analysis did not demonstrate a significant association between overall neonatal TPN exposure and either the primary composite outcome or T2DM. Accordingly, the association observed for the ≥3-day exposure category should be regarded as hypothesis-generating and should not be interpreted as evidence of a causal or biological threshold.

Future research should link administrative claims with neonatal medical records, detailed nutrition prescriptions, laboratory results, and longitudinal growth data. Such studies should evaluate cumulative energy exposure and the separate contributions of glucose, amino acids, lipid emulsions, and micronutrients, while accounting for illness severity and the timing of enteral feeding. Future analyses should also ensure clear temporal ordering of confounders, TPN initiation, and subsequent clinical events and consider time-varying exposure and confounding methods. External validation using duration-specific propensity weighting or other causal inference approaches would help determine whether the association observed for the ≥3-day category is reproducible or primarily reflects underlying neonatal illness severity.

## 5. Conclusions

The primary ATT-IPTW analysis showed no significant association between any neonatal TPN exposure and either the primary composite outcome or T2DM. In secondary exploratory analyses, TPN exposure for ≥3 days was associated with T2DM; however, this finding should be considered hypothesis-generating and may reflect residual confounding related to neonatal illness severity. This finding should not be interpreted as establishing a causal biological cutoff or as supporting the discontinuation of medically necessary TPN at 3 days. Rather, prolonged TPN exposure may serve as a marker of cumulative parenteral nutrient exposure and greater neonatal illness severity. Neonatal TPN should therefore remain available when clinically indicated, with regular reassessment of its ongoing necessity, advancement of enteral feeding when feasible, and individualized monitoring of infants requiring prolonged exposure. Further studies incorporating detailed nutrient composition and clinical severity data are needed to clarify the mechanisms underlying the observed duration-related association.

## Figures and Tables

**Table 1:** Baseline Characteristics Before and After IPTW (ATT).

Variable	TPN Group *n* = 7656	Control Group *n* = 2,295,911	Weighted Control Group Effective Sample Size *n* = 5301	SMD (Before)	SMD (After)
Mother age, y, mean (SD)	32.6 (4.3)	31.7 (4.0)	32.6 (4.2)	0.21	0.007
Household income level, *n* (%)					
Medical aid program	94 (1.2)	11,957 (0.5)	92 (1.7)	0.064	0.045
Q1 (lowest)	838 (10.9)	250,223 (10.9)	554 (10.4)	0.002	0.016
Q2	1790 (23.4)	561,125 (24.4)	1259 (23.7)	0.025	0.009
Q3	2837 (37.1)	868,716 (37.8)	1936 (36.5)	0.016	0.011
Q4 (highest)	1867 (24.4)	538,887 (23.5)	1260 (23.8)	0.021	0.014
Unknown	230 (3.0)	65,003 (2.8)	201 (3.8)	0.010	0.046
Residence, *n* (%)					
Metropolitan area	1918 (25.1)	574,086 (26.4)	1262 (24.8)		
Non-metropolitan area	5738 (74.9)	1,689,768 (73.6)	4039 (76.2)	0.031	0.029
Maternal History, *n* (%)					
Pregnancy-related neuropsychiatric disease	15 (0.2)	1122 (0.0)	9 (0.2)	0.033	0.008
Pre-gestational diabetes mellitus	642 (8.4)	122,627 (5.3)	452 (8.5)	0.11	0.005
Gestational diabetes mellitus	1216 (15.9)	286,584 (12.5)	836 (15.8)	0.093	0.003
Chronic hypertension	843 (11.0)	73,718 (3.2)	656 (12.4)	0.249	0.044
Preeclampsia/Eclampsia	1090 (14.2)	38,101 (1.7)	873 (16.5)	0.36	0.064
Hypertensive disorders of pregnancy	1386 (18.1)	121,979 (5.3)	1054 (19.9)	0.332	0.046
Pregnancy/Birth, *n* (%)					
Cesarean delivery	3675 (48.0)	776,146 (33.8)	2559 (48.3)	0.284	0.005
Birth asphyxia	844 (11.0)	9258 (0.4)	746 (14.1)	0.339	0.097
Adverse birth events	1542 (20.1)	43,288 (1.9)	1204 (22.7)	0.455	0.064
Neonatal factors, *n* (%)					
Female sex	3556 (46.4)	1,115,522 (48.6)	2465 (46.5)	0.043	0.001
Low birth weight or preterm	5879 (76.8)	54,405 (2.4)	4317 (81.4)	1.763	0.11
Bacterial sepsis	3611 (47.2)	56,673 (2.5)	2928 (55.2)	0.895	0.162
Necrotizing enterocolitis	564 (7.4)	1647 (0.1)	616 (11.6)	0.279	0.163
Fetal malnutrition	524 (6.8)	7619 (0.3)	484 (9.1)	0.258	0.091
Mechanical ventilation during NICU stay	5071 (66.2)	22,266 (1.0)	3855 (72.7)	1.38	0.137
Congenital circulatory malformations	1667 (21.8)	44,031 (1.9)	1466 (27.7)	0.481	0.143
Congenital GI malformations	878 (11.5)	48,761 (2.1)	1017 (19.2)	0.293	0.242
Pediatric CCC, *n* (%)					
0	4631 (60.5)	1,906,405 (83.0)	2890 (54.5)	0.461	0.122
1	2486 (32.5)	382,214 (16.6)	1816 (34.3)	0.338	0.038
≥2	539 (7.0)	7292 (0.3)	595 (11.2)	0.263	0.164
Year of baby birth, *n* (%)					
2005	601 (7.9)	360,680 (15.7)	467 (8.8)	0.29	0.034
2006	1005 (13.1)	374,246 (16.3)	747 (14.1)	0.094	0.029
2007	1275 (16.7)	412,457 (18.0)	838 (15.8)	0.035	0.023
2008	1542 (20.1)	386,304 (16.8)	1085 (20.5)	0.083	0.008
2009	1562 (20.4)	372,249 (16.2)	1084 (20.5)	0.104	0.001
2010	1667 (21.7)	389,975 (16.9)	1080 (20.3)	0.117	0.034

IPTW: Inverse probability of treatment weighting; ATT: Average treatment effect on the treated; CCC, complex chronic conditions; GI, gastrointestinal; NICU, neonatal intensive care unit; SD, standard deviation; SMD, standardized mean difference; TPN, total parenteral nutrition.

**Table 2:** Weighted HRs for metabolic or endocrinologic outcomes After IPTW (ATT).

Outcome	HR (95% CI)	*p*-Value
Any metabolic or endocrinologic disorder		
Control group	1	
TPN group	1.01 (0.94, 1.09)	0.801
Type 2 diabetes mellitus		
Control group	1	
TPN group	1.12 (0.93, 1.35)	0.215
Essential hypertension		
Control group	1	
TPN group	0.95 (0.68, 1.31)	0.733
Disorders of lipoprotein metabolism		
Control group	1	
TPN group	0.98 (0.91, 1.06)	0.615
Obesity		
Control group	1	
TPN group	1.02 (0.75, 1.39)	0.900
Metabolic syndrome		
Control group	1	
TPN group	0.95 (0.78, 1.15)	0.572

HRs were estimated using IPTW to adjust for all covariates listed in Table 1. IPTW: Inverse probability of treatment weighting; ATT: Average treatment effect on the treated; HR, hazard ratio; CI, confidence interval; TPN, total parenteral nutrition.

**Table 3:** Univariable and multivariable Cox regression analyses for endocrinologic and metabolic outcomes in the entire cohort.

Outcome	Univariable Analyses	*p*-Value	Multivariable Analyses	*p*-Value
	HR (95% CI)		HR (95% CI)	
Any metabolic or endocrinologic disorder				
TPN group (vs. control group)	1.68 (1.60, 1.76)	<0.001	0.98 (0.93, 1.03)	0.337
TPN, day	1.43 (1.38, 1.47)	<0.001	1.00 (0.96, 1.04)	0.960
TPN 1–2 days (vs. control group)	1.66 (1.58, 1.74)	<0.001	0.97 (0.92, 1.02)	0.284
TPN ≥ 3 days (vs. control group)	2.37 (1.83, 3.07)	<0.001	1.29 (1.00, 1.67)	0.055
Type 2 diabetes mellitus				
TPN group (vs. control group)	2.13 (1.92, 2.36)	<0.001	1.06 (0.95, 1.19)	0.311
TPN, day	1.66 (1.56, 1.76)	<0.001	1.12 (1.02, 1.21)	0.013
TPN 1–2 days (vs. control group)	2.07 (1.86, 2.30)	<0.001	1.04 (0.92, 1.16)	0.559
TPN ≥ 3 days (vs. control group)	4.61 (2.91, 7.32)	<0.001	2.14 (1.34, 3.41)	0.001
Essential hypertension				
TPN group (vs. control group)	3.34 (2.88, 3.88)	<0.001	1.00 (0.84, 1.18)	0.957
TPN, day	1.93 (1.79, 2.08)	<0.001	1.01 (0.89, 1.14)	0.921
TPN 1–2 days (vs. control group)	3.28 (2.82, 3.82)	<0.001	0.99 (0.83, 1.17)	0.879
TPN ≥ 3 days (vs. control group)	5.88 (2.80, 12.34)	<0.001	1.27 (0.60, 2.69)	0.527
Disorders of lipoprotein metabolism				
TPN group (vs. control group)	1.61 (1.53, 1.69)	<0.001	0.96 (0.91, 1.02)	0.191
TPN, day	1.39 (1.35, 1.44)	<0.001	0.99 (0.95, 1.04)	0.706
TPN 1–2 days (vs. control group)	1.59 (1.51, 1.67)	<0.001	0.96 (0.91, 1.01)	0.112
TPN ≥ 3 days (vs. control group)	2.31 (1.76, 3.03)	<0.001	1.30 (0.99, 1.71)	0.058
Obesity				
TPN group (vs. control group)	1.42 (1.15, 1.75)	0.001	0.84 (0.67, 1.05)	0.131
TPN, day	1.23 (1.05, 1.45)	0.011	0.84 (0.69, 1.03)	0.085
TPN 1–2 days (vs. control group)	1.45 (1.18, 1.79)	0.001	0.81 (0.65, 1.02)	0.067
TPN ≥ 3 days (vs. control group)	1.58 (1.20, 1.92)	<0.001	0.75 (0.49, 1.15)	0.184
Metabolic syndrome				
TPN group (vs. control group)	2.04 (1.84, 2.26)	<0.001	0.94 (0.84, 1.06)	0.321
TPN, day	1.63 (1.53, 1.73)	<0.001	1.02 (0.94, 1.12)	0.593
TPN 1–2 days (vs. control group)	1.99 (1.79, 2.21)	<0.001	0.93 (0.82, 1.04)	0.187
TPN ≥ 3 days (vs. control group)	4.17 (2.59, 6.70)	<0.001	1.69 (1.05, 2.73)	0.032

HR, hazard ratio; CI, confidence interval; TPN, total parenteral nutrition.

**Table 4:** Exploratory subgroup analyses for the primary composite outcome.

Outcome	Multivariable Analyses	*p*-Value
	HR (95% CI)	
Infants requiring mechanical ventilation support (*n* = 27,337)		
TPN group (vs. control group)	1.11 (1.04, 1.19)	0.002
TPN, day	1.09 (1.04, 1.15)	<0.001
TPN 1–2 days (vs. control group)	1.10 (1.03, 1.18)	0.005
TPN ≥ 3 days (vs. control group)	1.46 (1.11, 1.92)	0.006
Infants with LBW or fetal growth restriction (*n* = 63,023)		
TPN group (vs. control group)	1.05 (0.98, 1.12)	0.153
TPN, day	1.07 (1.02, 1.12)	0.009
TPN 1–2 days (vs. control group)	1.04 (0.97, 1.10)	0.293
TPN ≥ 3 days (vs. control group)	1.59 (1.19, 2.12)	0.002
Infants admitted to the neonatal NICU (*n* = 47,242)		
TPN group (vs. control group)	0.99 (0.93, 1.06)	0.819
TPN, day	1.02 (0.97, 1.07)	0.371
TPN 1–2 days (vs. control group)	0.98 (0.92, 1.04)	0.527
TPN ≥ 3 days (vs. control group)	1.60 (1.21, 2.12)	0.001

HR, hazard ratio; CI, confidence interval; TPN, Total Parenteral Nutrition; NICU, neonatal intensive care unit.

## Data Availability

Data will be available upon reasonable request to corresponding author.

## Supplementary Materials

The following supporting information can be downloaded at: https://www.mdpi.com/article/10.3390/nu18152406/s1, Figure S1: Spearman correlogram of neonatal TPN duration and long-term endocrinologic or metabolic outcomes. Supplementary File S1. Detailed Definitions and ICD-10 Codes for Study Variables. Supplementary File S2. All HRs with 95% CIs in multivariable Cox regression model for Endocrinologic and Metabolic Disorders.

## Abbreviations

The following abbreviations are used in this manuscript:

CI	Confidence interval
DOHaD	Developmental Origins of Health and Disease
ESPEN	European Society for Clinical Nutrition and Metabolism
HR	Hazard ratio
ICD-10	International Classification of Diseases, 10th Revision
IPTW	Inverse probability of treatment weighting
LBW	Low birth weight
NHIS	National Health Insurance Service
NICU	Neonatal intensive care unit
T2DM	Type 2 diabetes mellitus
TPN	Total parenteral nutrition
