# Supplementary material for: Neonatal Total Parenteral Nutrition and Long-Term Metabolic Outcomes: An 18-Year Nationwide Cohort Study of 2.3 Million Infants
Source: Nutrients. 2026 Jul 23;18(15):2406. doi: 10.3390/nu18152406 (PMC13468498; doi:10.3390/nu18152406)
Supplement: Supplementary file 1 [file nutrients-18-02406-s001.zip › Supplementary File S1.pdf]

Supplementary File S1. Detailed Definitions and ICD-10 Codes for Study Variables

1. Maternal Sociodemographic and Clinical Covariates

- **Socioeconomic Status:** Categorized by household income quintiles (Q1–Q4) and participation in the government-subsidized Medical Aid program. Residence was dichotomized into metropolitan and non-metropolitan areas based on administrative districts.
- **Pre-existing Medical Conditions:**
    - **Pre-existing (chronic) hypertension:** Essential or secondary hypertension diagnosed prior to pregnancy (ICD-10 codes I10–I15).
    - **Pre-gestational diabetes mellitus:** Type 1 or Type 2 diabetes diagnosed before the index pregnancy (ICD-10 codes E10–E14).
    - **Asthma or allergic rhinitis**: Chronic inflammatory disorders of the respiratory tract or nasal mucosa, often driven by IgE-mediated hypersensitivity (ICD-10 codes J45, J30).
    - **Atopic dermatitis**: A chronic, relapsing inflammatory skin disease characterized by pruritus and a strong association with other atopic conditions (ICD-10 codes L20).
    - **Autoimmune thyroid disease**: Chronic autoimmune inflammation of the thyroid gland, most commonly presenting as Hashimoto's thyroiditis (ICD-10 codes E06.3).
    - **Rheumatoid arthritis**: A systemic autoimmune disease primarily manifesting as chronic, erosive inflammation of the synovial joints (ICD-10 codes M05, M06).
    - **Ankylosing Spondylitis**: A type of chronic inflammatory arthritis that primarily affects the axial skeleton, including the sacroiliac joints and spine (ICD-10 codes M45).
    - **Systemic lupus erythematosus**: A multi-system autoimmune disease involving the production of antinuclear antibodies and diverse organ involvement (ICD-10 codes

M32).

- **Inflammatory bowel disease**: Chronic idiopathic inflammation of the gastrointestinal tract, encompassing both Crohn's disease and ulcerative colitis (ICD-10 codes K50, K51).
- **Psoriasis**: An immune-mediated chronic inflammatory skin condition resulting in hyperproliferation of keratinocytes and scaly plaques (ICD-10 codes L40).
- **Type 1 Diabetes Mellitus**: A condition characterized by absolute insulin deficiency due to the autoimmune destruction of pancreatic beta cells (ICD-10 codes E10).
- **Neurological or mental disorders**: Mental disorders and diseases of the nervous system that complicate the clinical management of pregnancy and childbirth (ICD-10 codes O99.3).

2. Pregnancy and Delivery-related Factors

- **Obstetric Complications:**
  - **Hypertensive disorders of pregnancy:** Including gestational hypertension and related syndromes (ICD-10 codes O10–O16).
  - **Gestational diabetes mellitus:** Onset of glucose intolerance during the second or third trimester (ICD-10 code O24).
- **Delivery Profile:** Mode of delivery was identified as Cesarean section (National Health Insurance procedure codes R4501–R4521) or vaginal delivery.

3. Neonatal Clinical Characteristics

- **Perinatal Morbidities:**
  - **Fetal growth and nutrition:** Including fetal malnutrition and small-for-gestational-age status (ICD-10 code P05) and preterm or low birth weight status (ICD-10 code

P07).

  - **Perinatal distress:** Birth asphyxia (ICD-10 code P21), respiratory distress syndrome (ICD-10 codes P22.0) and other adverse birth-related events.
  - **Infectious and gastrointestinal conditions:** Bacterial sepsis of the newborn (ICD-10 code P36) and necrotizing enterocolitis (ICD-10 code P77).
- **Critical Care Interventions:** Mechanical ventilation during neonatal intensive care unit stay was identified through billing codes for invasive or non-invasive respiratory support.
- **Neonatal intensive care unit admission**

---

4. Pediatric Complex Chronic Conditions (CCC) The baseline health status of the child was further characterized using Pediatric Complex Chronic Condition categories (Version 2) based on the following ICD-10 codes:

- **Neuromuscular**: Conditions affecting the central or peripheral nervous system (G10–G13, G20–G26, G30–G32, G35–G37, G40–G41, G70–G73, G80–G83, F70–F79, Q00–Q07).
- **Cardiovascular**: Congenital or acquired disorders of the heart (I00–I02, I05–I09, I11, I20–I25, I26–I28, I30–I52, Q20–Q28).
- **Respiratory**: Chronic conditions of the lungs and airways (J40–J47, J60–J70, J95–J99, Q30–Q34).
- **Gastrointestinal**: Severe disorders of the digestive tract requiring specialized management (K20–K31, K50–K52, K70–K77, K80–K87, Q38–Q45).
- **Renal**: Chronic diseases of the kidneys and urinary tract (N00–N08, N10–N16, N17–N19, Q60–Q64).
- **Metabolic**: Inborn errors of metabolism and endocrine disorders (E00–E07, E10–E14, E70–

E88, Q86–Q89).

- **Hematologic**: Diseases of the blood and blood-forming organs (D50–D53, D55–D59, D60–D64, D65–D69, D70–D77, D80–D89).
- **Congenital/Genetic**: Chromosomal abnormalities and malformations not organ-specific (Q10–Q18, Q65–Q79, Q80–Q85, Q90–Q99).
