# Supplementary material for: Neonatal Total Parenteral Nutrition and Long-Term Metabolic Outcomes: An 18-Year Nationwide Cohort Study of 2.3 Million Infants
Source: Nutrients. 2026 Jul 23;18(15):2406. doi: 10.3390/nu18152406 (PMC13468498; doi:10.3390/nu18152406)
Supplement: Supplementary file 1 [file nutrients-18-02406-s001.zip › Supplementary File S2.pdf]

Supplementary File S2. All HRs with 95% CIs in multivariable Cox regression model for Endocrinologic and Metabolic Disorders

Variable	HR (95% CI)	*P*-value
Mother age, y	1.01 (1.01, 1.01(	<0.001
Household income level		
Medical aid program	1.43 (1.38, 1.48)	<0.001
Q1 (lowest)	1	
Q2	0.98 (0.97, 0.99)	<0.001
Q3	0.99 (0.98, 0.99)	0.023
Q4 (highest)	1.02 (1.01, 1.03)	<0.001
Unknown	1.13 (1.11, 1.15)	<0.001
Residence		
Metropolitan area	1	
Non-metropolitan area	1.16 (1.16, 1.17)	<0.001
Maternal History		
Pregnancy-related neuropsychiatric disease	0.98 (0.93, 1.03)	0.417
Pre-gestational diabetes mellitus	1.13 (1.11, 1.22)	<0.001
Gestational diabetes mellitus	1.18 (1.17, 1.19)	<0.001
Chronic hypertension	1.02 (1.01, 1.04)	0.003
Preeclampsia/Eclampsia	1.03 (1.01, 1.06)	0.001
Hypertensive disorders of pregnancy	1.02 (1.01, 1.03)	<0.001
Pregnancy/Birth		
Cesarean delivery	1.11 (1.10, 1.12)	<0.001
Birth asphyxia	1.43 (1.38, 1.48)	<0.001
Adverse birth events	1.65 (1.61, 1.68)	<0.001
Neonatal factors		

Female sex	1.05 (1.04, 1.05)	<0.001
Low birth weight or preterm	1.32 (1.26, 1.38)	<0.001
Bacterial sepsis	1.16 (1.14, 1.19)	<0.001
Necrotizing enterocolitis	0.80 (0.73, 0.87)	<0.001
Fetal malnutrition	1.29 (1.26, 1.31)	<0.001
Mechanical ventilation during NICU stay	1.19 (1.15, 1.22)	<0.001
Congenital circulatory malformations	1.11 (1.09, 1.13)	<0.001
Congenital GI malformations	1.17 (1.14, 1.19)	<0.001
Pediatric CCC		
0	1	
1	0.84 (0.83, 0.84)	<0.001
≥ 2	0.92 (0.88, 0.95)	<0.001

HR, hazard ratio; CI, confidence interval; CCC, complex chronic conditions; GI, gastrointestinal; NICU, neonatal intensive care unit; TPN, total parenteral nutrition.
